# Supplementary material for: MariClus: Your One-Stop Platform for Information on Marine Natural Products, Their Gene Clusters and Producing Organisms
Source: Mar Drugs. 2023 Aug 15;21(8):449. doi: 10.3390/md21080449 (PMC10455768; doi:10.3390/md21080449)
Supplement: Supplementary file 1 [file marinedrugs-21-00449-s001.zip › marinedrugs-2519661-supplementary.pdf]

MariClus: your one-stop platform for information on marine natural products, their gene clusters and producing organisms

Cedric Hermans [1,†], Maarten L. De Mol [2,†], Marieke Mispelaere [1], Anne-Sofie De Rop [2], Jeltien Rombaut [2], Tesneem Nusayr [3], Rebecca Creamer [4], Sofie L. De Maeseneire [2], Wim K. Soetaert [2] and Paco Hulpiau [1,*]

[1] Bioinformatics Knowledge Center (BiKC), Campus Brugge Station, Howest University of Applied Sciences, Rijselstraat 5, 8200 Bruges, Belgium
[2] Centre for Industrial Biotechnology and Biocatalysis (InBio.be), Department of Biotechnology, Faculty of Bioscience Engineering, Ghent University, Coupure Links 653, 9000 Ghent, Belgium
[3] Life Sciences, Texas A&M-Corpus Christi, TX. 78412, USA
[4] Entomology, Plant Pathology, and Weed Science, New Mexico State University, Las Cruces, NM 88003, USA
* Correspondence: maarten.demol@ugent.be; paco.hulpiau@howest.be.
† These authors contributed equally to this work.

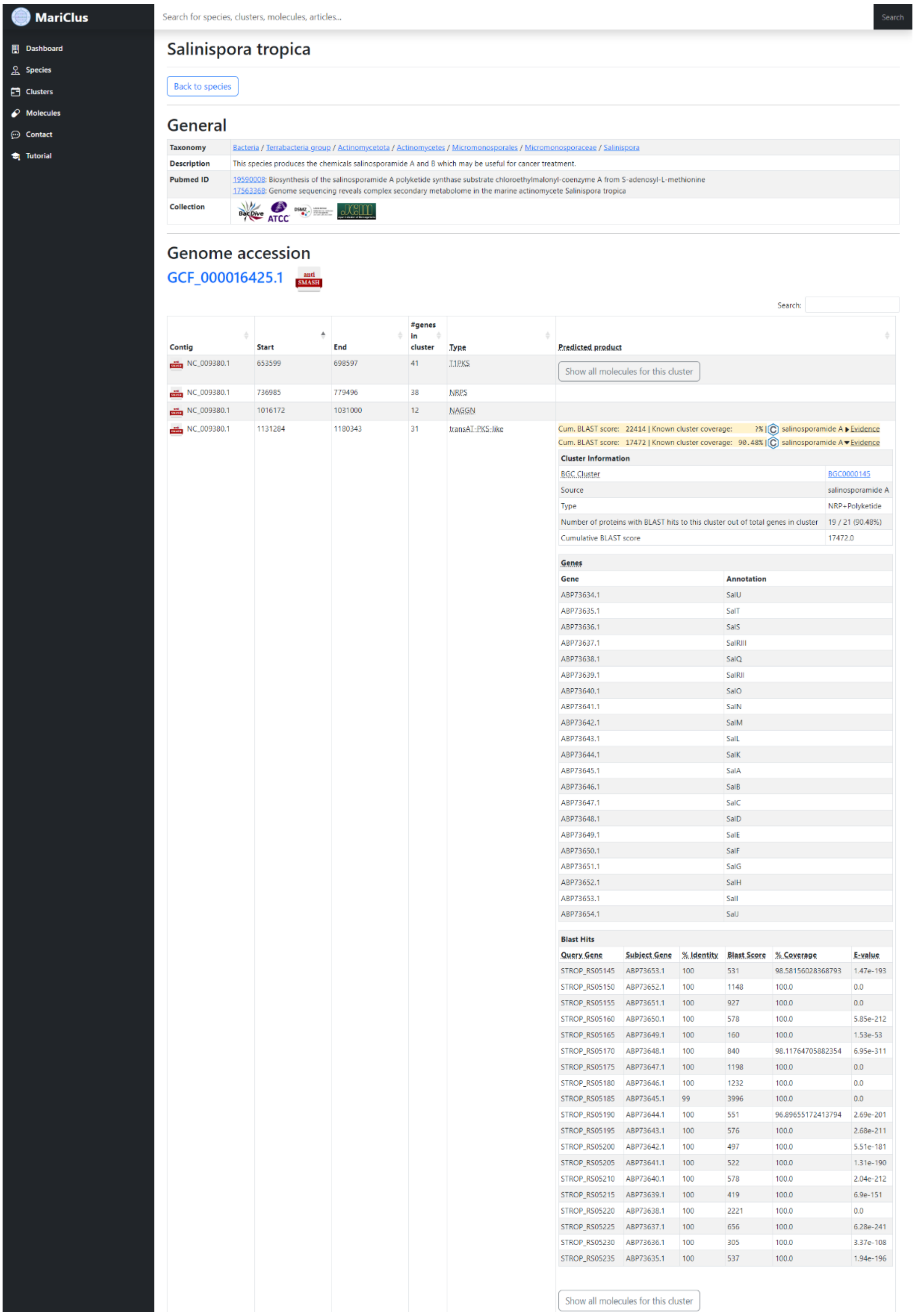

Supplementary Figure S1. MariClus database view of the species details for *Salinispora tropica*. The page shows the information about the species, such as its taxonomic lineage, genome assembly, publications and culture collection number. It also shows the list of clusters and molecules predicted for the species. The molecule Salinosporamide A is highlighted in the table as this was our search query. The evidence for the second occurrence of the molecule in this species has been expanded, showing more information about the corresponding MIBiG cluster, the number of BLAST hits and the scores of each hit to the MIBiG cluster. The user can access the antiSMASH pages of the cluster and the molecule by clicking on the icons. The view is limited to the clusters of interest.

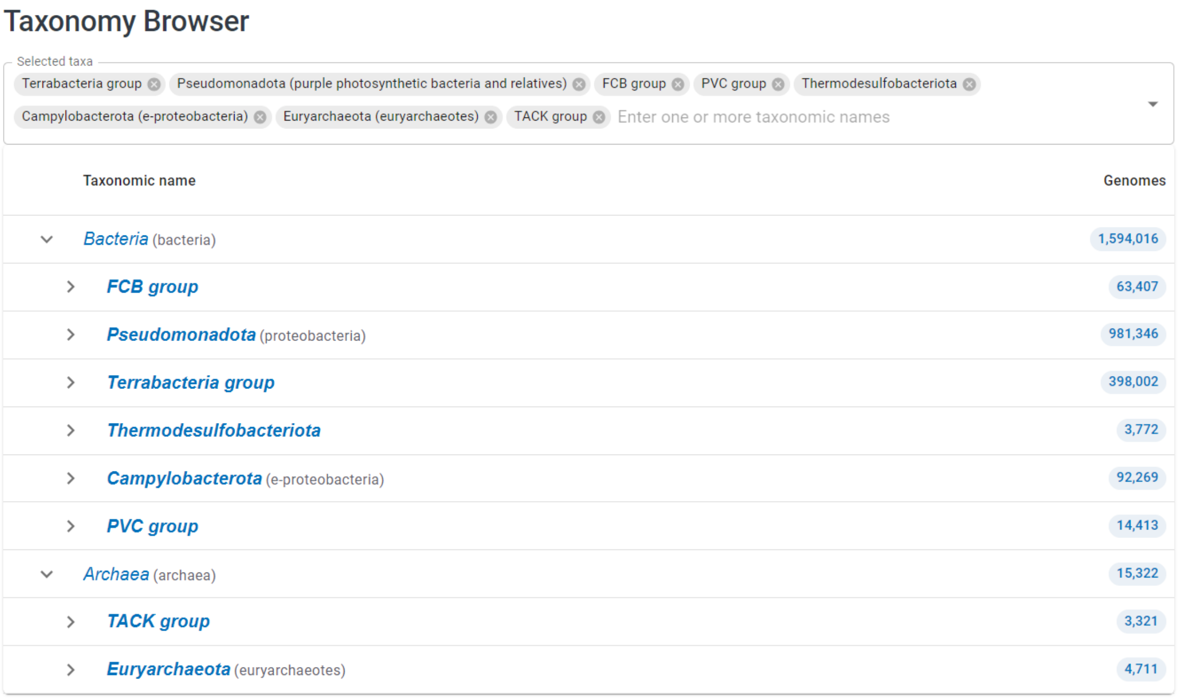
Taxonomy Browser

Selected taxa: Terrabacteria group, Pseudomonadota (purple photosynthetic bacteria and relatives), FCB group, PVC group, Thermodesulfobacteriota, Campylobacterota (e-proteobacteria), Euryarchaeota (euryarchaeotes), TACK group. Enter one or more taxonomic names

Taxonomic name	Genomes
Bacteria (bacteria)	1,594,016
FCB group	63,407
Pseudomonadota (proteobacteria)	981,346
Terrabacteria group	398,002
Thermodesulfobacteriota	3,772
Campylobacterota (e-proteobacteria)	92,269
PVC group	14,413
Archaea (archaea)	15,322
TACK group	3,321
Euryarchaeota (euryarchaeotes)	4,711

Supplementary Figure S2. NCBI taxonomy browser results for the top 8 phyla of the MariClus database. The table shows the taxonomic names and the number of genomes in each phylum. The Pseudomonadota and Terrabacteria group together account for more than 80% of all entries in the bacteria superkingdom (Sayers *et al*. 2022)[16].

References

Sayers, Eric W., Evan E. Bolton, J. Rodney Brister, Kathi Canese, Jessica Chan, Donald C. Comeau, Ryan Connor, et al. 2022. “Database Resources of the National Center for Biotechnology Information.” *Nucleic Acids Research* 50 (D1): D20–26. https://doi.org/10.1093/nar/gkab1112.
